# Effect of two prophylaxis methods on marginal gap of Cl Vresin-modified glass-ionomer restorations

**DOI:** 10.15171/joddd.2016.004

**Published:** 2016-03-16

**Authors:** Soodabeh Kimyai, Fatemeh Pournaghi-Azar, Mehdi Daneshpooy, Mehdi Abed Kahnamoii, Farnaz Davoodi

**Affiliations:** ^1^Dental and Periodontal Research Center, Faculty of Dentistry, Tabriz University of Medical Sciences, Tabriz, Iran; ^2^Professor, Department of Operative Dentistry, Faculty of Dentistry, Tabriz University of Medical Sciences, Tabriz, Iran; ^3^Assistant Professor, Department of Operative Dentistry, Faculty of Dentistry, Tabriz University of Medical Sciences, Tabriz, Iran; ^4^Associate Professor, Department of Operative Dentistry, Faculty of Dentistry, Tabriz University of Medical Sciences, Tabriz, Iran; ^5^Post-graduate student, Department of Operative Dentistry, Faculty of Dentistry, Tabriz University of Medical Sciences, Tabriz, Iran

**Keywords:** Dental marginal adaptation, dental prophylaxis, glass-ionomer cements

## Abstract

***Background.*** This study evaluated the effect of two prophylaxis techniques on the marginal gap of CI V resin-modified glass-ionomer restorations.

***Methods.*** Standard Cl V cavities were prepared on the buccal surfaces of 48 sound bovine mandibular incisors in this in vitro study. After restoration of the cavities with GC Fuji II LC resin-modified glass-ionomer, the samples were randomly assigned to 3 groups of 16. In group 1, the prophylactic procedures were carried out with rubber cup and pumice powder and in group 2 with air-powder polishing device (APD). In group 3 (control), the samples did not undergo any prophylactic procedures. Then the marginal gaps were measured. Two-way ANOVA was used to compare marginal gaps at the occlusal and gingival margins between the groups. Post hoc Tukey test was used for two-by-two comparisons. Statistical significance was set at P < 0.05.

***Results.*** There were significant differences in the means of marginal gaps in terms of prophylactic techniques (P < 0.001), with significantly larger marginal gaps in the APD group compared to the pumice and rubber cup group, which in turn exhibited significantly larger marginal gaps compared to the control group (P < 0.0005). In addition, the means of marginal gaps were significant in terms of the margin type (P < 0.001), with significantly larger gaps at gingival margins compared to the occlusal margins (P < 0.0005).

***Conclusion.*** The prophylactic techniques used in this study had a negative effect on the marginal gaps of Cl V resin-modified glass-ionomer restorations.

## Introduction

Prophylactic techniques used to remove plaque and stains from tooth surfaces might increase surface roughness of restorations and compromise their surface polish, resulting in increased bacterial adhesion, plaque accumulation, gingival inflammation and recurrent caries, depending on duration of use and the specific technique used. Cervical restorations are more susceptible to the destructive effects of prophylactic techniques due to higher plaque and stain retention in that area of teeth.^1,2^

Various studies have evaluated the effect of different prophylactic techniques on the surface roughness of a variety of tooth-colored dental restorative materials, reporting that the effect of these techniques depends on the type of the restorative material.^1-4^ Soares et al^5^ evaluated the samples under an electron microscope and reported that prophylaxis with air-powder polishing device (APD) results in surface porosity and roughness and gap formation at indirect composite resin restoration-tooth interface, while prophylaxis with pumice results in grooves on the surface of the tooth and indirect composite resin restorations. In addition, a study on the effects of different prophylactic techniques on the microleakage of CI V cavities restored with microfilled composite resin showed that prophylactic measures (APD, pumice powder with rubber cup, and pumice powder with brush) had no detrimental effect on the microleakage of cavities.^6^ In this context, another study showed that cleaning with ultrasound and APD did not increase marginal microleakage of amalgam and composite resin restorations.^7^ However, another study showed that prophylaxis with APD, compared with pumice and rubber cup, increased the surface roughness of giomer restorative material.^8^ In addition, Rajstein et al^9^ reported that ultrasound cleaning with the use of a scaler had a deleterious effect on the surface of class V amalgam restorations, with a destructive effect on the marginal integrity of these restorations. Destruction of the marginal integrity, followed by microleakage, might give rise to tooth discoloration, postoperative sensitivity, recurrent caries, and pulp inflammation.^10,11^

Resin-modified glass-ionomer is a tooth-colored restorative material used for the restoration of cervical cavities.^12^ In addition to its high mechanical properties and esthetic appearance, this material has the potential to release fluoride, which has resulted in its widespread use for the restoration of cervical cavities in patients with a high risk for caries.^12,13^

Considering the importance of marginal integrity of restorations in periodontal-restorative interaction,^6^ and since no studies to date have evaluated the effect of prophylactic techniques on the marginal gaps of resin-modified glass-ionomer restorations, the aim of the present study was to evaluate the effect of two different prophylactic techniques (APD vs. pumice + rubber cup) on the marginal gaps of Cl V resin-modified glass-ionomer restorations.

## Methods

The study protocol was approved by the Ethics Committee at Tabriz University of Medical Sciences (Ref. No. 125) and the bovine teeth were collected according to a protocol approved by the Regional Medical Research Ethics Committee.

This *in vitro* study was carried out on 48 sound permanent bovine mandibular incisors, with no cracks, fractures, anomalies, and defects as evidenced by visual inspection and evaluation under a stereomicroscope (SMZ1500, Nikon, Tokyo, Japan). The tooth samples were immersed in a 0.5% chloramine-T trihydrate bacteriostatic/bactericidal solution (Merck KGaA, Darmstadt, Germany) for a week, followed by storage in distilled water in a refrigerator at a temperature of 4ºC. The storage medium was renewed at regular intervals. Twenty-four hours before the experimental procedures, the teeth were immersed and conditioned in distilled water at a temperature of 23±2ºC.

Cl V cavities were prepared on the buccal surfaces of teeth, with occluso-gingival and mesiodistal dimensions of 3×3 mm and a depth of 2 mm.^6^ The occlusal wall was placed 1.5 mm coronal to CEJ and the gingival wall was placed 1.5 mm apical to CEJ. The cavities were prepared with a diamond fissure bur (Diatech Dental AG, Swiss Dental Instruments, CH-9435 Heerbrugg) in a high-speed handpiece under air and water cooling. The bur was replaced with a new one after every five cavity preparation procedures. All the cavity margins were butt joint without any bevels.^6^ During preparation, tooth surfaces were kept wet to avoid dehydration.

The cavity conditioner (GC Corporation, Tokyo, Japan) which contains 20% polyacrylic acid was applied to the bonding surfaces for 10 seconds according to manufacturer’s instructions, to remove the smear layer and condition the enamel and dentin. After irrigation and gentle drying, the powder and fluid of Fuji II LC resin-modified glass-ionomer restorative material (GC Corporation, Tokyo, Japan) were mixed according to manufacturer’s instructions (1 level scoop of powder to 2 drops of liquid) and placed in the cavities. A transparent matrix band (Hawe Neos Dental, Bioggio, Switzerland) was placed on the restorative material and light-curing was carried out with a halogen light-curing unit (Astralis 7, Ivoclar Vivadent, FL-9494 Schaan, Liechtenstein) at a light intensity of 400 mW/cm^2^ with the light-conducting tip just touching and perpendicular to the surface for 20 seconds. Post-curing was carried out for‏ 60 seconds at an output power of 700 mW/cm^2^‏. The restorative procedures were carried out by one operator. After the restorative procedures and removal of the matrix band, the samples were polished with diamond finishing burs (Diamont Gmbh, D&Z, Berlin, Germany) and polishing disks (Sof-Lex, 3M ESPE, Dental Products,‏ St. Paul, MN, USA).

Subsequently, the specimens underwent a storage procedure in distilled water at 37ºC for a 24-hour period. In order to simulate the oral cavity conditions, the samples underwent a 500-round thermocycling procedure at 5±2/55±2ºC, with a 30-second dwell time and a 10-second transfer time. The specimens underwent a storage procedure in distilled water at 37ºC for a period of three months in order to simulate inter-appointment periods during the maintenance phase in the clinic.^3^

Then the samples were randomly divided into 3 groups (n=16). In group 1, the sample surfaces underwent a prophylactic procedure with slurry of pumice (Kemdent, Swindon, Wiltshire, UK) and a rubber cup (Stoddard, Letchworth, Hertfordshire, UK) for 120 seconds using a slow-speed contra-angle handpiece at 2000 rpm. A separate rubber cup was used for each sample.

In group 2, the sample surfaces underwent a prophylactic procedure using APD (Air-Flow, Electronic Medical Systems, Nyon, Switzerland) for 120 seconds at a distance of 10 mm from the sample surface and perpendicular to it, during which regular powder was used during the first 60 second and fine powder during the second 60 minutes. All the preparation procedures were carried out by one operator. Then the samples in groups 1 and 2 were rinsed with water and placed in an ultrasonic bath for 10 minute for further cleaning of the surfaces.^1,2^ No prophylactic procedures were carried out in group 3 (the control). Finally, the teeth were sectioned in a buccolingual direction at the middle of the restorations with the use of a diamond disk (Diamont Gmbh, D&Z, Berlin, Germany). Gap widths were measured‏ at occlusal and gingival margins under a stereomicroscope (SMZ1500, Nikon, Tokyo, Japan) at ×40.^14^ A digital camera was used to photograph the selected areas with the use of a DS-L2 control unit (Nikon, Tokyo, Japan) so that the gaps could be measured. The gap widths were measured with the built-in software by determining a tangential line on the tooth-side vector and by measuring the distance between the points on restoration-side vector and the line mentioned above. These measurement techniques were repeated at three locations: outer, middle and inner portions of the occlusal and gingival margins. In each group, the mean marginal gap widths at the three‏ locations mentioned above were calculated in micrometers.

Two-way ANOVA was used for the analysis of data in the study groups with SPSS 20.0. Post hoc Tukey test was used for the two-by-two comparisons of the groups. Statistical significance was set at P<0.05.

## Results

**Figure 1.**
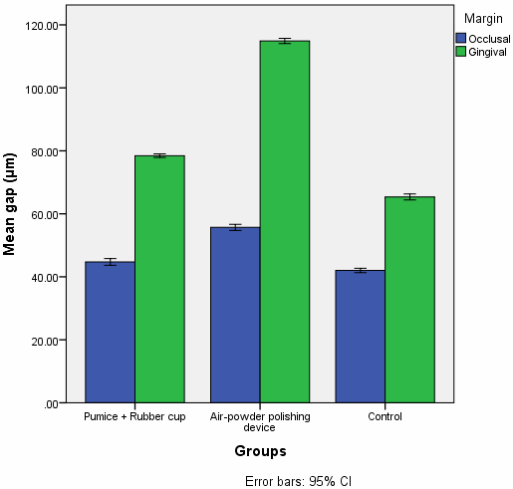
The bar graph with error bars of mean marginal gap widths in the study groups.

Table 1 presents the descriptive data on marginal gaps and the results of comparisons made between the study groups. Figure 1 presents the error bars of the means of marginal gaps in the study groups.

**Table 1:** The means and standard deviations of marginal gap widths (in µm) in the study groups

**Groups**	**Marginal gap width (µm)**	
	**Occlusal**	**Gingival**
**Prophylaxis with pumice and rubber cup**	44.72±1.99^a^	78.45±1.09^b^
**Prophylaxis with air-powder polishing device**	55.70±1.82^c^	114.89±1.57^d^
**Without prophylaxis (control)**	42.01±1.31^e^	65.34±1.76^f^

Mean values with different letters indicate statistically significant differences in analysis with post hoc Tukey test.

The results of two-way ANOVA showed significant differences in the mean marginal gap widths in terms of the prophylactic technique applied (F_2,90_ = 3282.78, P<0.001). In addition, post hoc Tukey test showed significant differences between the two prophylactic techniques (P<0.001). In this context, in the APD prophylactic technique group, marginal gaps were significantly wider than those in the group undergoing a prophylactic procedure with pumice and rubber cup (P<0.0005); the marginal gaps in the pumice and rubber cup prophylactic group were significantly wider than those in the control group (with no prophylactic procedures) (P<0.0005).

In addition, the means of marginal gap widths were significantly different in terms of the margin type (F_1,90_ = 13657.95, P<0.001), with significantly larger gaps at gingival margins compared to the occlusal margins (P<0.0005). In addition, the interaction between the type of the prophylactic technique and the margin type was statistically significant (F_2,90_ = 1031.72, P<0.001).

## Discussion

It is of utmost importance to preserve the marginal seal and prevent disruption of marginal integrity during the clinical service of restorations in restorative dentistry. Disruption of marginal seal and an increase in the gap widths of a restoration might give rise to marginal discoloration, recurrent caries, hypersensitivity, pain, and demineralization along the cavity margins.^15,16^ The aim of the present study was to evaluate the effect of two different prophylactic techniques (pumice with rubber cup and APD) on the marginal gaps of Cl V resin-modified glass-ionomer restorations. The results showed significantly wider marginal gaps in these restorations in groups undergoing prophylactic procedures compared to the control group. In addition, prophylaxis with APD resulted in significantly wider marginal gaps.

Wider marginal gaps in groups undergoing prophylactic procedures, compared to the control group, might be attributed to destruction of surface polishing of resin-modified glass-ionomer at margins with the use of prophylactic techniques. Glass-ionomer is a heterogeneous and biphasic material in nature. The set material is composed of non-reactive glass particles embedded in a polysalt resin matrix. During prophylactic procedures, each abrasive particle functions as a fine tool and creates a groove on the surface of the restorative material. The softer matrix phase of glass-ionomer is selectively removed and non-reactive glass particle remains and protrudes from the surface. Destruction of the restorative material might lead to an increase in surface roughness, margin degradation, disruption of the material structure and even debonding of the restorative material,^3^ the two latter of which can increase the marginal gap width.

In a study by Wu et al,^3^ application of APD to resin-modified glass-ionomer resin resulted in more degradation of the surface polish and in more surface roughness compared to the group in which no prophylactic procedures were carried out; however, there were no significant differences in surface roughness between the pumice + rubber cup group and the control group; nevertheless, in the present study, in both prophylactic procedures there were significantly wider marginal gaps compared to the control group. Such a discrepancy between the results might be attributed to the different methodology of the mentioned study in which the surface roughness was evaluated. Another finding of the present study was the fact that APD resulted in more marginal gaps compared to the pumice and rubber cup, which might be due to the higher water and air pressure in the APD procedure, which results in better elimination of the resin matrix of resin-modified glass-ionomer. It has even been reported that in the APD technique the powder particles can abrade the filler phase of composite resin materials.^4^ In relation to resin-modified glass-ionomer, further studies are required to evaluate whether it is possible for non-reactive glass particles to be abraded by APD particles.

In relation to the deleterious effect of prophylactic procedures on restorations, a study by Rajstein et al,^9^ showed that ultrasound scaling has a negative effect on the marginal integrity of Cl V amalgam restorations, resulting in the widening of marginal gaps of these restorations. It has been reported that sonic and ultrasonic scalers result in chips, scratches and loss of restorative materials.^17^ Salami et al^18^ reported that use of APD resulted in abrasion of root surfaces and destruction of restorations and resin cements. Soares et al^5^ using electron microscope evaluations reported that APD destroyed the cement line in indirect composite resin restorations and produced a rough and porous surface. In addition, it formed gaps at the restoration‒tooth interface. However, prophylaxis with pumice and rubber cup only produced grooves on the surface of the tooth and the restorative materials (indirect composite resin) and did not lead to gap formation. Nonetheless, in the present study, both APD and pumice-and-rubber-cup techniques resulted in more gap formation compared to the control group. One of the reasons for the difference between the results of the latter study,^5^ and those of ours might be the nature of the evaluated materials and their surface characteristics. The resistance of tooth-colored restorative materials to abrasion might be influenced by the type of the resin, the size and percentage of the fillers, and the polymerization technique used.^19^ It has been reported that the abrasion resistance of resin-modified glass-ionomer is less than that of composite resins.^20^ It has been shown in another study that compared to resin composites, conventional and resin-modified glass-ionomers are more susceptible to surface changes with the use of sonic and ultrasonic periodontal instrumentation.^1^

Contrary to the results of the present study, Gorifil et al^7^ did not find prophylaxis with APD and ultrasonic scaler to result in an increase in marginal leakage and disruption of marginal integration of Cl V composite resin and amalgam restorations. In addition, in a study by Kimyai et al,^6^ the prophylactic techniques evaluated (APD, pumice with rubber cup, and pumice with brush) did not result in an increase in marginal microleakage of Cl V microfilled composite resin restorations. The discrepancies among the mentioned study results might be attributed to differences in the studied materials and their resistance to abrasion, the measuring techniques, and the instrumentation parameters.^21^ Previous studies have not reported any relationship between marginal gap and leakage.^22,23^

Another finding of the present study was the fact that marginal gasps were present in all groups, even in the control samples, with significantly wider marginal gaps at gingival (dentinal) margins compared to the occlusal (enamel) margins in all study groups. Gap formation in the control group might be attributed to the polymerization shrinkage of resin-modified glass-ionomer and the stresses resulting from that.^24^ One study has reported stresses from polymerization shrinkage or expansion due to water sorption within the resin-modified glass-ionomer results in adhesive bond failure and formation of cracks in dentin.^25^

Presence of wider gaps at gingival margins compared to occlusal margins might be attributed to the homogeneous structure of enamel and better adhesion to enamel structure, while adhesion to dentin is more complex due to its non-homogeneous structure, movement of fluids toward the external surface of dentin, and lower inorganic content in dentin.^26^

The results of the present study are consistent with those of previous studies, in which it was shown that microleakage at Cl II and Cl V sandwich restorations with resin-modified glass-ionomer is significantly higher at dentinal margins compared to enamel margins.^27^ In addition, in a study by Wilder et al,^28^ Cl V resin-modified glass-ionomer restorations exhibited more microleakage at dentinal margins compared to enamel margins.

Contrary to the results of the present study, Chuang et al^29^reported that marginal seal in Cl V resin-modified glass-ionomer restorations (in un-etched groups compared to etched groups) was significantly more favorable at dentinal margins compared to enamel margins. The reason for the discrepancy between the results of the present study and those of the latter study might be differences in methodology, including the number of rounds in the thermocycling procedure. In the study of Chuang et al,^29^ thermocycling procedure, consisting of 1500 rounds, was carried out to simulate the aging process. Enamel has a high coefficient of elasticity and dentin has a low coefficient of elasticity. It has been reported that with long-term storage in water, resin-modified glass-ionomer undergoes volumetric expansion. Therefore, the dentin beneath the restoration has a higher capacity to absorb stress to buffer the expansion of the material. Enamel cracks have been reported adjacent to resin-modified glass-ionomer during thermocycling, which might result in microleakage.^30^

In addition, in a study by Farmer et al,^31^ resin-modified glass-ionomer exhibited more microleakage in Cl V cavities at enamel margins than that in dentinal margins, compared to composite resin. The authors attributed this difference to the hydrophilic nature of glass-ionomer compared to composite resin.

In a study by Toledano et al,^32^ Cl V resin-modified glass-ionomer restorations did not exhibit any significant differences in marginal gaps between enamel and dentinal margins. Another study showed resin-modified glass-ionomer to have similar bonding ability to enamel and dentin.^33^

Several factors affect the marginal integrity of the restorations, including the type of substrate, such as the type of the restorative material and the structure of enamel or dentin, and the experimental conditions. Therefore, there are wide variations in data in different studies, depending on the applied technique and the manipulative variables used during the placement of bonded materials. In addition, marginal gaps might be influenced by the physicochemical properties of the materials.^22^

As a future line of research, it is recommended that the effect of various prophylactic methods on marginal gaps of different types of glass-ionomer and other tooth-colored restorative materials be evaluated and also electron microscopy be used for the analysis of marginal gaps.

## Conclusion

Under the limitations of the present *in vitro* study, both prophylactic methods resulted in an increase in marginal gaps of resin-modified glass-ionomer restorations.

## Acknowledgements

The authors would like to thank Dr. M. Abdolrahimi, DDS, who edited the English manuscript of this article.

## Authors’ contributions

The study was planned by SK, FP and MAK. MD and FD carried out the experimental studies. MAK performed the gap measurements. The statistical analyses and interpretation of data was made by SK and FP who also prepared the first draft. All authors contributed to the final draft and approved the final manuscript.

## Funding

This study was supported by a grant from Dental and Periodontal Research Center at Tabriz University of Medical Sciences.

## Competing interests

The authors declare that they have no competing interests with regards to authorship and/or publications of this paper.

## Ethics approval

The study protocol was approved by the Ethics Committee at Tabriz University of Medical Sciences (Ref. No. 125) and the bovine teeth were collected according to a protocol approved by the Regional Medical Research Ethics Committee.
